# Inhibition of HDAC3 Ameliorates Cerebral Ischemia Reperfusion Injury in Diabetic Mice In Vivo and In Vitro

**DOI:** 10.1155/2019/8520856

**Published:** 2019-02-13

**Authors:** Bo Zhao, Quan Yuan, Jia-bao Hou, Zhong-yuan Xia, Li-ying Zhan, Mei Li, Meng Jiang, Wen-wei Gao, Lian Liu

**Affiliations:** ^1^Department of Anesthesiology, Renmin Hospital of Wuhan University, Wuhan, Hubei Province 430060, China; ^2^Department of Critical Care Medicine, Renmin Hospital of Wuhan University, Wuhan, Hubei Province 430060, China

## Abstract

**Background:**

A substantial increase in histone deacetylase 3 (HDAC3) expression is implicated in the pathological process of diabetes and stroke. However, it is unclear whether HDAC3 plays an important role in diabetes complicated with stroke. We aimed to explore the role and the potential mechanisms of HDAC3 in cerebral ischemia/reperfusion (I/R) injury in diabetic state.

**Methods:**

Diabetic mice were subjected to 1 h ischemia, followed by 24 h reperfusion. PC12 cells were exposed to high glucose for 24 h, followed by 3 h of hypoxia and 6 h of reoxygenation (H/R). Diabetic mice received RGFP966 (the specific HDAC3 inhibitor) or vehicle 30 minutes before the middle cerebral artery occlusion (MCAO), and high glucose-incubated PC12 cells were pretreated with RGFP966 or vehicle 6 h before H/R.

**Results:**

HDAC3 inhibition reduced the cerebral infarct volume, ameliorated pathological changes, improved the cell viability and cytotoxicity, alleviated apoptosis, attenuated oxidative stress, and enhanced autophagy in cerebral I/R injury model in diabetic state in vivo and in vitro. Furthermore, we found that the expression of HDAC3 was remarkably amplified, and the Bmal1 expression was notably decreased in diabetic mice with cerebral I/R, whereas this phenomenon was obviously reversed by RGFP966 pretreatment.

**Conclusions:**

These results suggested that the HDAC3 was involved in the pathological process of the complex disease of diabetic stroke. Suppression of HDAC3 exerted protective effects against cerebral I/R injury in diabetic state in vivo and in vitro via the modulation of oxidative stress, apoptosis, and autophagy, which might be mediated by the upregulation of Bmal1.

## 1. Introduction

Stroke is one of the leading causes of death and disability worldwide. In the past 20 years, stroke has been the top three in the leading causes of death in China's major diseases, and ischemic stroke accounts for 87% of all strokes [1]. At present, tissue plasminogen activator (t-PA) for thrombolysis is the only established treatment for ischemic stroke in clinic. However, the time window (3-4.5 hours) is so narrow that only a small proportion (3-5%) of patients are eligible [2]. Diabetes increases the vulnerability and susceptibility of brain vessels, which constitute a major risk factor for ischemic stroke [3]. Moreover, stroke has become one of the leading causes of death in diabetic patients in China [4, 5]. It is estimated that more than 415 million people worldwide had diabetes in 2015, and the number is projected to increase to 642 million by 2040 [6]. Diabetic patients in China is estimated to increase from 20.8 million in 2000 to 42.3 million by 2030 [7]. Diabetic patients are more likely to suffer stroke than nondiabetic patients, the prognosis is worse, and the mortality rate is higher after stroke [8]. Epidemiological surveys indicated that diabetes increases the risk of stroke by twofold to fivefold [9]. In China, prevalence of stroke in the patients with diabetes accounts for more than 5.5% [10]. Unfortunately, the classical treatment of stroke by t-PA thrombolysis in diabetics induces an increased incidence of intracerebral hemorrhage and worse neurological outcomes compared with nondiabetic population [11]. Furthermore, some new and potential therapies that benefit nondiabetic stroke patients have failed to translate successfully into diabetic stroke counterparts [12]. Therefore, it is of great clinical significance to explore the pathogenesis and effective therapeutic strategies for diabetes complicated with stroke.

Epigenetic modifications play key roles in the pathophysiology of multiple diseases and are the current research hotspot [13]. Histone acetylation and deacetylation are major participants in epigenetic changes. Histone acetyltransferases (HATs) act on histones to promote chromatin release, thereby stimulating transcription and activating gene expression. Histone deacetylases (HDACs) act as another modifier to promote histone deacetylation in lysine (Lys, K) residues, thereby inhibiting transcription and gene expression. The level of histone acetylation is determined by the dynamic balance between HATs and HDACs [14]. Typically, HDACs act as transcriptional inhibitors to silence gene expression and induce chromatin compaction [15]. Thus, inhibition of HDACs can alter the above balance and help to enhance histone acetylation, chromatin relaxation, and gene expression [16]. Since many small molecules can effectively regulate HDAC activity and exert therapeutic potentials for various diseases (including metabolic disorders and cerebrovascular diseases) [17], it is of great importance to understand the role of individual HDACs in the pathophysiology of diseases.

Histone deacetylase 3 (HDAC3), a member of the HDAC family, is expressed in almost all human tissues, including the brain, and has been reported to be upregulated in diabetes and cerebral ischemic stroke [18–20]. Depletion of the HDAC3 in local tissue, such as skeletal muscle, can cause severe systemic insulin resistance in mice [21]. Epigenetic intervention of HDAC3 with lentiviral vectors (shHDAC3) led to an enormous reduction in cell survival. Mice are reported to die within 2 days after receiving shHDAC3-infected grafts [22]. But evidences suggest that selective inhibition of HDAC3 may be an attractive strategy for targeting diabetes and may increase the resistance of the central nervous system to ischemic injury [23, 24]. However, the key mechanism of HDAC3 in diabetic ischemic stroke remains unclear. The brain and muscle Arnt-like 1 (Bmal1), a core component of the circadian clock, is highly expressed in the brain and negatively regulated by HDAC3. Other than regulating the circadian rhythm, Bmal1 is involved in the regulation of adipogenesis, glucose homeostasis, and lipid metabolism. Bmal1 has also been proven to be implicated in the regulation of oxidative stress and autophagy, which are highly related to the pathologies of various diseases. The involvement of HDAC3 and Bmal1 in diabetic ischemic stroke needs further clarification.

Based on the above background, in the present study, by establishing the model of diabetes and cerebral ischemia/reperfusion (I/R) injury in vivo and in vitro, we initially investigated whether inhibition of HDAC3 reduces cerebral I/R injury in diabetic mice. Diabetes has been proven to increase oxidative stress and impair autophagy in the brain and exacerbate cerebral ischemic injury in animal models [25–27]. It is well known that oxidative stress plays a fundamental role in the pathogenesis of transient cerebral ischemic injury [28]. Autophagy dysfunction is an important pathophysiological characteristic during I/R injury [29]. Therefore, testing levels of oxidative stress and autophagy may indicate whether inhibition of HDAC3 is vital for diabetic stroke. More importantly, we further explored the underlying mechanisms of diabetes combined with ischemic stroke, hoping to find a new target for this complex disease.

## 2. Materials and Methods

### 2.1. Animals

Thirty-six adult male C57BL/6 mice (20-22 g) were purchased from HFK Bioscience Co. Ltd. (Beijing, China). All mice were housed in the Animal Center of Renmin Hospital of Wuhan University in a standard environment and with a 12-hour light/12-hour dark cycle. All experimental protocols were approved by the Medical Faculty Ethics Committee of Wuhan University, and experimental processes were performed according to the National Institutes of Health *Guide for the Care and Use of Laboratory Animals*. Eight weeks after STZ injection, the mice were randomly divided into three groups: mice underwent sham operation group (DS), mice subjected to I/R injury (DIR), and mice pretreated with RGFP966 and subjected to I/R injury (DIR-H). HDAC3-specific inhibitor RGFP966 was subcutaneously injected with 10 mg/kg 30 min before middle cerebral artery occlusion (MCAO). The concentration of RGFP966 was determined based on the previous research [30].

### 2.2. Reagents

Streptozotocin (STZ) and triphenyl tetrazolium chloride (TTC) were provided by Sigma Chemical Co. (MO, USA). The HDAC3 inhibitor RGFP966 was obtained from Selleck Chemicals Co. (Texas, USA). The Dulbecco's modified Eagle's medium (DMEM), fetal bovine serum (FBS), and bovine serum albumin (BSA) were purchased from Gibco Laboratories (Grand Island, NY, USA). Antibodies directed against HDAC3, Bmal1, beclin-1, and autophagy-related proteins—microtubule-associated protein 1 light chain 3 B (LC3B), sequestosome 1 (p62), and GAPDH—were acquired from Cell Signaling Technology (CST, Beverly, CA, USA).

### 2.3. Cell Culture

PC12 cells (the neuron-like rat pheochromocytoma cell line) were obtained from the China Center for Type Culture Collection (CCTCC) and were cultured in DMEM containing 10% FBS, 100 U/ml penicillin, and 100 U/ml streptomycin and maintained in a humidified incubator with 5% CO_2_ at 37°C. Cell medium was replaced every other day, and cells were incubated at an appropriate density according to experimental scale. The concentration of glucose in DMEM was 25 mm. PC12 cells were randomly assigned to three groups: high glucose control (the HG group), cells exposed to high glucose followed by hypoxia/reoxygenation (H/R) (the HH/R group), and cells pretreated with RGFP966 and exposed to high glucose followed by H/R (the HH/R-H group). H/R was induced by exposing cells to hypoxia (95% N_2_ and 5% CO_2_) for 3 h, followed by reoxygenation of 6 h. RGFP966 (10 *μ*mol/L) was treated 6 h before H/R. The concentration of RGFP966 was determined based on the previous research and the manufacturers' instructions [30].

### 2.4. Diabetes Induction

After 7 days of acclimation to the environment, the mice were starved for 12 hours for diabetes induction. Diabetes was induced by intraperitoneal injection of 1% STZ (50 mg/kg). STZ was dissolved in 0.1 mol/L sodium citrate buffer (pH = 4.5). After 72 h (with 6 h fasting), blood samples were obtained from the tail vein to evaluate the blood glucose using OneTouch Ultra Glucometer (Johnson & Johnson, USA). If the fasting blood glucose was ≥16.7 mmol/L at least for the three samples, the mice were considered to have diabetes.

### 2.5. Cerebral Ischemia/Reperfusion (I/R) Injury Model Construction

Focal cerebral ischemia was induced by MCAO in the mice using a silk plug, as previously described [31]. Eight weeks after diabetes induction, the mice were deeply anaesthetized with isoflurane, and the left external carotid artery (ECA) was exposed. The silk plug was inserted into the middle cerebral artery branch of the ECA. The suture was carefully removed after being placed in the middle cerebral artery for 60 min. The success of the model is marked by the presence of hemiparalysis, which is heavier in the ischemic contralateral limb after the anesthesia regains. The neurological score was measured by a blinded investigator at 24 hours after MCAO according to a neurological grading scale previously described [31]: 0, asymptomatic; 1, right forelimb cannot be straightened when lifting the tail; 2, rotating to the right side when walking; 3, pour to the right side when walking; and 4, no spontaneous activity and/or level of consciousness was depressed. The mice that showed a neurological score with 1 to 4 and brains with no detectable hemorrhage were included for analysis. Subsequently, six mice in each group were randomly selected for TTC staining, and the rest six mice in each group were decapitated, and the brain tissues were cryopreserved or fixed for follow-up biochemical detection.

### 2.6. 2,3,5-Triphenyltetrazolium Chloride (TTC) Staining

Cerebral infarct volume was detected by TTC staining. The six mice of each group were sacrificed to assess the infarct volume, as described previously [31]. The brain was placed into a special slice mold for the mice brain and was cut into 2 mm thick coronal sections. The sections were soaked in TTC (Sigma, USA) for 30 min, stained, and immersed in 4% paraformaldehyde for overnight fixation. The slices are arranged neatly and scanned in layers. The infarct volume was determined by a blinded observer and corrected for edema by using the ImageJ software (version 1.61; National Institutes of Health, Bethesda, MD).

### 2.7. Haematoxylin and Eosin Staining (H&E Staining)

After 24 h of reperfusion, the six mice of each group were anesthetized and decapitated, and their brains were fixed with 4% formaldehyde solution for 24 h, embedded in paraffin, and cut into 4 *μ*m slices by a microtome. The slices were stained with haematoxylin and eosin stain (H&E). The H&E stain was quantified under the BX51 microscope (×400, Olympus Inc., Japan).

### 2.8. Reactive Oxygen Species (ROS), Malondialdehyde (MDA), and Superoxide Dismutase (SOD) Measurement

Mitochondria-generated ROS was measured using the commercial kits (Nanjing Jiancheng Bio-Engineering Institute, China). Samples from different groups were incubated in a reaction buffer; malate and glutamate were added as substrates. After 40 min incubation at 37°C, the formation of the oxidized fluorescent product dichlorofluorescein was monitored with excitation at 488 nm and emission at 525 nm. The results were expressed as arbitrary fluorescence units per mg protein (U/mg). The MDA content and SOD activity were also detected using the corresponding kits (Nanjing Jiancheng Bio-Engineering Institute, China) according to the manufacturers' instructions. Briefly, brain tissues or PC12 cells were collected and homogenized in 10 vol (*w*/*v*) ice-cold 0.1 M PBS. Then, the homogenate was centrifuged at 4000×*g* for 10 min, and the supernatant was used for detection of MDA level and SOD activity. The precision of the assay for MDA and SOD was defined by calculating the intra- and interassay coefficient of variation (CV).

### 2.9. TUNEL Staining

The cerebral apoptosis rate was determined by terminal deoxynucleotidyl transferase dUTP nick end labeling assay (TUNEL). Paraffin-embedded sections were dewaxed and rehydrated and then incubated in 20 *μ*L/mL proteinase K for 15 min. TUNEL was accomplished using an in situ cell death detection kit (Roche Inc., USA). After immersion in an equilibration buffer for 10 min, sections were incubated with TdT and dUTP-digoxigenin, then washed before incubation in antidigoxigenin-peroxidase solution, and finally colored with diaminobenzidine-H_2_O_2_ solution. The number of TUNEL-positive cells was quantified under the BX51 microscope (×400, Olympus Inc., Japan).

### 2.10. Flow Cytometry

Apoptotic cells were determined by flow cytometry. After reoxygenation, cells were collected and resuspended in a binding buffer and were incubated with Annexin V-PE and 7-aminoactinomycin D (7-AAD) stains for 10 min in the dark. Cellular fluorescence was measured using the FACSCalibur instrument (BD Biosciences, USA). The data obtained from the cell population were analysed using the CellQuest Pro software (BD Biosciences, USA). Cells located in the right two quadrants of each plot were considered as apoptotic cells, and the percentages of apoptosis were determined by flow cytometry.

### 2.11. Immunofluorescence Staining

The brain tissues were deparaffinized, dehydrated with gradient alcohol, and then antigen repaired. The sections were washed with 0.01 M PBST for 5 min × 3 times and placed in 10% BSA wet box (37°C) for 30 min. The primary antibodies (beclin-1 and LC3B: diluted 1 : 100, respectively) were added and incubated overnight at 4°C and then added secondary fluorescent antibody (diluted 1 : 100) and incubated at room temperature for 1 hour in wet box, rinsing with 0.01 M PBST in the dark for 5 min × 3 times. Finally, the images under the fluorescence microscope were collected after being sealed by a glycerol buffer.

### 2.12. Transmission Electron Microscopy (TEM) Observation

TEM was performed to observe autophagic vacuole ultrastructure. Brain tissue samples (approximately 1 mm^3^) were fixed with 2.5% glutaraldehyde, dehydrated with gradient alcohol and acetone, embedded in epoxy resin to prepare ultrathin sections, and double-stained with uranyl acetate-lead citrate. The slices were finally observed and photographed under a TEM (Hitachi, Japan) at 5000 (5 K) magnification.

### 2.13. Cell Counting Kit-8 (CCK-8) and Lactate Dehydrogenase (LDH) Release Assay

Cell viability was determined by the CCK-8 assay, according to the manufacturer's instructions. PC12 cells were cultured in 6-well plates and pretreated with various conditions (HG, HH/R, and HH/R-H) as described previously, following which 10 *μ*L CCK-8 (C0037, Beyotime, China) was added and cells were incubated for 4 hours, and the absorbance at 450 nm was measured using a microplate reader. The mean optical density (OD) of 6 wells in each group was used to calculate the percentage of cell viability. The supernatant was collected to measure the effluent LDH after PC12 cells performed above pretreatment. LDH release was measured by LDH Cytotoxicity Assay Kit (Nanjing Jiancheng Bio-Engineering Institute, China).

### 2.14. Western Blotting Analysis

The brain tissue (20 mg) or cells were homogenized and lysed using the RIPA buffer containing protease inhibitor mixture (Beyotime). The homogenates were centrifuged at 12000g at 4°C for 15 min, supernatants were collected, and total protein concentration was determined using a bicinchoninic acid (BCA) kit (Beyotime). Equivalent amounts of proteins (20 *μ*g) were loaded into each lane and separated by 10% SDS gels and then transferred onto PVDF membrane. The membrane was blocked with 5% BSA for 1 h and incubated with the following primary rabbit monoclonal antibodies: HDAC3, Bmal1, and p62 (1 : 1000, Cell Signaling Technology, USA) diluted in 5% *w*/*v* BSA overnight at 4°C. The following day, the HRP-labelled secondary antibody (anti-rabbit, 1 : 10000, Millipore, USA) was incubated for 1 h and then washed with TBST for chemiluminescence development. The ImageJ software (version 1.61; National Institutes of Health, Bethesda, MD) was used to quantify the Western blot bands.

### 2.15. Statistical Analysis

All data were expressed as mean ± SD and analysed by SPSS 17.0 software (SPSS, Chicago, IL, USA). One-way analysis of variance (ANOVA) was used for statistical comparisons between the different groups. *P* < 0.05 was considered to be statistically significant.

## 3. Results

### 3.1. HDAC3 Inhibition Exerted Protective Effects against Cerebral I/R Injury in Diabetic State

**Figure 1:**
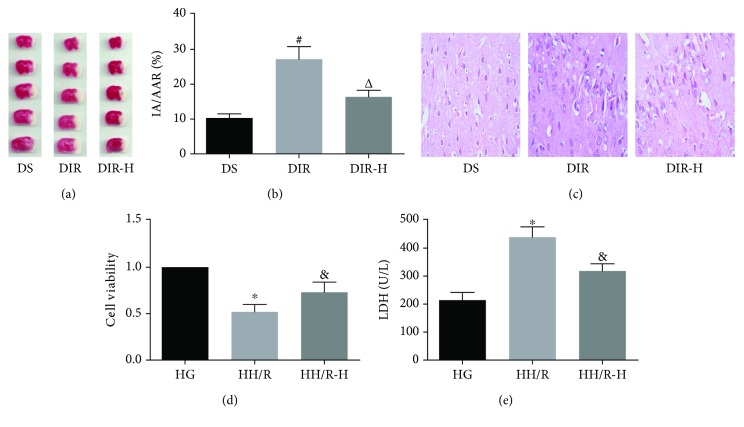
Inhibition of HDAC3 played protective effects against cerebral I/R injury. Brain tissues were collected for determination of cerebral infarct volume by TTC staining (a, b) and observation of pathological changes by H&E staining (c) in each group. PC12 cells were collected for assessment of cell viability assessed by CCK-8 (d) and cytotoxicity by lactate dehydrogenase (LDH) release (e) in each group. All values are presented as mean ± SD, *n* = 6/group. ^#^*P* < 0.05 versus the DS group, ^△^*P* < 0.05 versus the DIR group, ^∗^*P* < 0.05 versus the HG group, and ^&^*P* < 0.05 versus the HH/R group.

The HDAC3 inhibitor decreased the cerebral infarct volume and ameliorated pathological changes of the brain tissues in the diabetic mice subjected to I/R injury. As shown in Figures 1(a) and 1(b), the cerebral infarct volume in the DIR group was significantly increased compared with that in the DS group (DIR versus DS, *P* < 0.05). RGFP966 is a well-known HDAC3-specific inhibitor and has been proven to be effective in the brain. Administration of RGFP966 in the DIR-H group significantly decreased the infarct volume compared with that in the DIR group (DIR-H versus DIR, *P* < 0.05). Next, we used H&E staining to evaluate the pathological changes in brain tissues (Figure 1(c)). Microscopically, we found that the neuronal structures were damaged, the cytoplasm was unevenly distributed, vacuoles were formed, and the nuclei were condensed in the DIR group when compared with those in the DS group. In the DIR-H group, the structures were basically restored to normal, most of the neurons were intact, some cytoplasm vacuolated, and the nuclei were relatively clear relative to the DIR group. In in vitro study, the HDAC3 inhibitor improved the viability and cytotoxicity of PC12 cells impaired by HH/R (Figures 1(d) and 1(e)). Following 3 h of hypoxia and 6 h of reoxygenation, cell viability was significantly reduced, and cellular LDH release was increased in the HH/R group (HH/R versus HG, *P* < 0.05). However, when cells were incubated with RGFP966 in the HH/R-H group, HH/R-induced reduction of cell viability and elevation of cellular LDH release were significantly ameliorated (HH/R-H versus HH/R, *P* < 0.05).

### 3.2. HDAC3 Inhibition Alleviated I/R-Induced Apoptosis in Diabetic State

**Figure 2:**
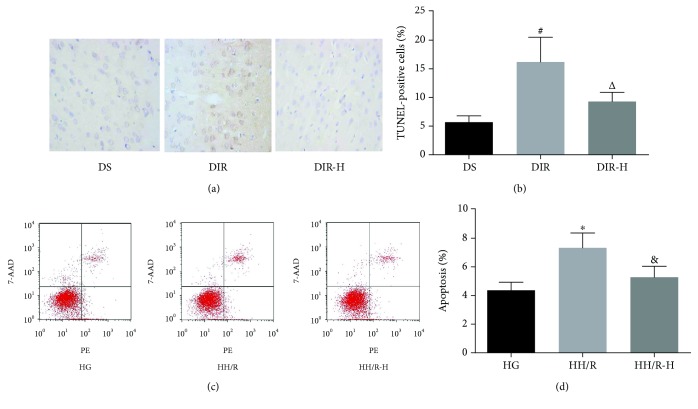
Inhibition of HDAC3 alleviated I/R-induced apoptosis. Apoptosis in the brain tissues was assessed by TUNEL staining (a, b); in the PC12 cells, it was evaluated by flow cytometry (c, d) in each group. All values are presented as mean ± SD, *n* = 6/group. ^#^*P* < 0.05 versus the DS group, ^△^*P* < 0.05 versus the DIR group, ^∗^*P* < 0.05 versus the HG group, and ^&^*P* < 0.05 versus the HH/R group.

To examine whether HDAC3 inhibitor administration alleviates I/R-induced cell apoptosis in diabetic state, we evaluated the apoptosis rate by the TUNEL assay (Figures 2(a) and 2(b)), further conformed by the flow cytometric analysis (Figures 2(c) and 2(d)). In vivo, apoptotic cells increased in the DIR group (DIR versus DS, *P* < 0.05). While pretreated with RGFP966, the number of TUNEL-positive cells in the DIR-H group was significantly lower than that in the DIR group (DIR-H versus DIR, *P* < 0.05). In vitro, PC12 cells exposed to high glucose and H/R showed a prominent increase in apoptosis compared with the HG group (HH/R versus HG, *P* < 0.05). Similarly, cells pretreated with RGFP966 remarkably decreased the apoptosis in the HH/R-H group when compared with those in the HH/R group (HH/R-H versus HH/R, *P* < 0.05).

### 3.3. HDAC3 Inhibition Attenuated I/R-Induced Oxidative Stress in Diabetic State

**Figure 3:**
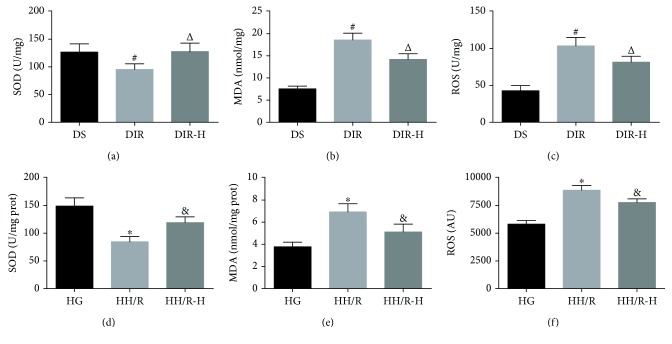
Inhibition of HDAC3 attenuated I/R-induced oxidative stress. Brain tissues were collected for analysis of SOD activity (a), MDA content (b), and ROS levels (c); PC12 cells were also collected for detection of SOD activity (d), MDA content (e), and ROS levels (f). All values are presented as mean ± SD, *n* = 6/group. ^#^*P* < 0.05 versus the DS group, ^△^*P* < 0.05 versus the DIR group, ^∗^*P* < 0.05 versus the HG group, and ^&^*P* < 0.05 versus the HH/R group.

To evaluate the effects of HDAC3 inhibition on oxidative stress in this study, we measured the levels of ROS generation, the MDA content, and SOD activity (Figure 3). ROS is considered to be the major cause of tissue injury after cerebral ischemia. As a final product of lipid peroxidation, MDA is a widely accepted indicator for oxidative stress [32]. SOD, a major member of the antioxidant defense system, is another important indicator of oxidative stress [33]. For MDA content and SOD activity, the intra-assay and interassay coefficient of variation (CV) values were all <15%. SOD activity (Figure 3(a)) was significantly decreased, and the levels of MDA (Figure 3(b)) and ROS (Figure 3(c)) were prominently increased in the brain tissues of the DIR group compared with those in the DS group (DIR versus DS, *P* < 0.05). RGFP966 in the DIR-H group markedly increased the SOD activity (Figure 3(a)) and decreased the levels of MDA (Figure 3(b)) and ROS (Figure 3(c)) in the DIR-H group compared to the DIR group (DIR-H versus DIR, *P* < 0.05). The results were verified in the PC12 cells in vitro. Compared to the HG group, the SOD activity (Figure 3(d)) decreased, and the MDA content (Figure 3(e)) and the ROS production (Figure 3(f)) increased in the HH/R group (HH/R versus HG, *P* < 0.05). However, the activity of SOD (Figure 3(d)) increased, and the levels of MDA (Figure 3(e)) and ROS (Figure 3(f)) decreased when RGFP966 was used in the HH/R-H group as compared with those in the HH/R group (HH/R-H versus HH/R, *P* < 0.05).

### 3.4. HDAC3 Inhibition Enhanced Autophagy in Diabetic Mice Subjected to I/R

**Figure 4:**
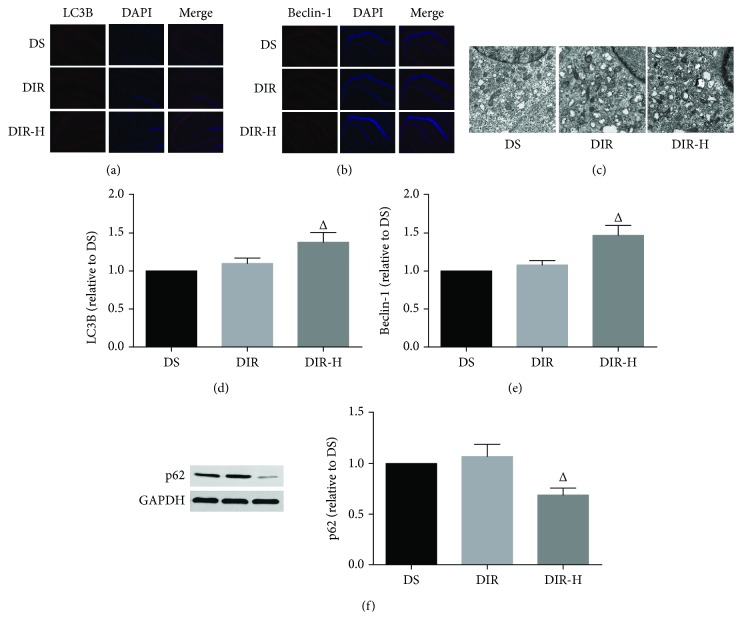
HDAC3 inhibition enhanced autophagy in diabetic mice subjected to cerebral I/R injury. Representative immunofluorescence staining images and assessment of LC3B (a, d) and beclin-1 (b, e) (×100). Representative electron micrographs of each group (c) (×5000). Representative Western blot images and assessment of p62 (f) level in each group. All results are presented as mean ± SD, *n* = 6/group. ^#^*P* < 0.05 versus the DS group and ^△^*P* < 0.05 versus the DIR group.

In order to estimate the autophagy alterations, autophagy-related proteins (LC3B, beclin-1, and p62) were detected (Figure 4). LC3B is a widely used marker for autophagosome, and beclin-1 is an essential autophagy protein known to induce autophagy [34]. As the autophagy-specific substrate, the protein levels of p62 are inversely related to autophagic activity [35]. In this study, the expressions of LC3B (Figures 4(a) and 4(d)) and beclin-1 (Figures 4(b) and 4(e)) were assessed by immunofluorescence, and p62 (Figure 4(f)) was determined by Western blotting. Compared to the DS group, I/R slightly increased the expression of beclin-1 and LC3B and slightly decreased the expression of p62 in the DIR group (DIR versus DS, *P* > 0.05). However, the expressions of beclin-1 and LC3B were remarkably increased, and p62 was markedly decreased after RGFP966 was applied in the DIR-H group (DIR-H versus DIR, *P* < 0.05). Then, TEM was used to detect autophagic vacuoles, which are generally formed in cells undergoing the autophagic process. Thus, autophagic vacuoles inside the cells are an indicator to evaluate the induction extent of autophagy. As shown in Figure 4(c), brain samples from the DIR group subjected to I/R injury displayed a slight increase in the number of the autophagosomes, which were characterized by bubble-like vacuoles enclosing cytoplasmic material and/or organelles relative to the DS group. However, the appearance of organelles was less damaged, and more autophagosomes were generated after application with RGFP966 in the DIR-H group than that in the DIR group.

### 3.5. The Protective Effects of RGFP966 Were Mediated by the Upregulation of Bmal1

**Figure 5:**
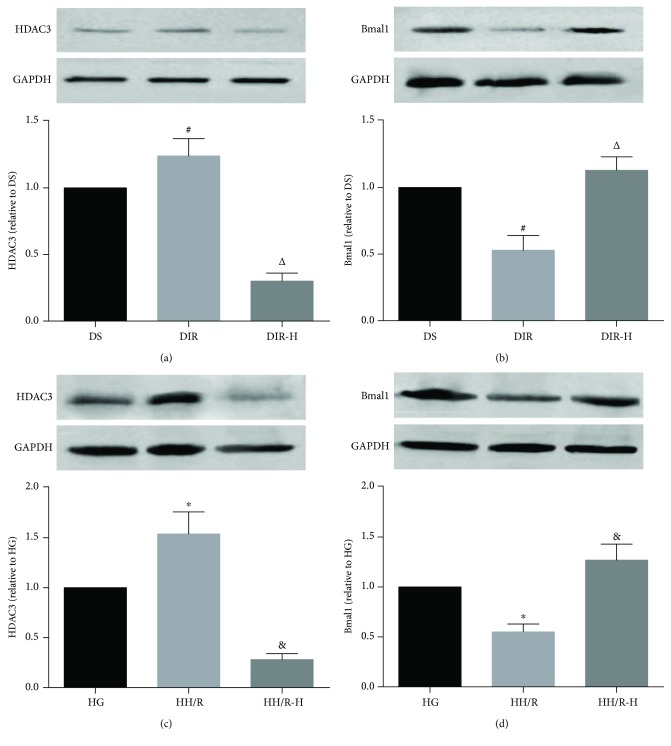
The protective effects of RGFP966 were mediated by the upregulation of Bmal1. Representative Western blot images and assessment of the HDAC3 and Bmal1 level in each group. HDAC3 and Bmal1 expression in the mouse brain (a, b) and HDAC3 and Bmal1 expression in PC12 cells (c, d). All results are presented as mean ± SD, *n* = 6/group. ^#^*P* < 0.05 versus the DS group, ^△^*P* < 0.05 versus the DIR group, ^∗^*P* < 0.05 versus the HG group, and ^&^*P* < 0.05 versus the HH/R group.

After evaluating the roles of HDAC3 in cerebral I/R in diabetic state, we tried to determine the underlying mechanism of HDAC3 in cerebral I/R in vivo and in vitro. The expressions of HDAC3 (Figures 5(a) and 5(c)) and Bmal1 (Figures 5(b) and 5(d)) were determined by Western blotting. The diabetic mice subjected to cerebral I/R significantly increased the expression of HDAC3, while decreased the expression of Bmal1 in the DIR group compared to the DS group (DIR versus DS, *P* < 0.05). Notably, HDAC3 expression was remarkably downregulated by RGFP966 administration, which led to the upregulation of Bmal1 in the diabetic mice subjected to cerebral I/R (DIR-H versus DIR, *P* < 0.05). Then, we confirmed the results in PC12 cells exposed to high glucose. The HDAC3 expression was prominently increased, and the Bmal1 expression was markedly decreased after H/R in the HH/R group relative to the HG group (HH/R versus HG, *P* < 0.05). However, when PC12 cells were incubated with RGFP966 in the HH/R-H group, the HDAC3 expression was significantly downregulated, and the Bmal1 expression was notably upregulated (HH/R-H versus HH/R, *P* < 0.05).

## 4. Discussion

In the present study, we have demonstrated that the inhibition of HDAC3 by RGFP966, a highly selective inhibitor with an excellent blood-brain barrier penetration [30], significantly reduced the cerebral infarct volume, ameliorated pathological changes, improved the cell viability and cytotoxicity, alleviated apoptosis, attenuated oxidative stress, and enhanced autophagy in cerebral I/R injury model in diabetic state in vivo and in vitro. Furthermore, we found that the expression of HDAC3 was remarkably amplified, and the Bmal1 expression was notably decreased in the diabetic mice with cerebral I/R injury, whereas this phenomenon was obviously reversed by RGFP966 pretreatment. To our knowledge, this is the first study to investigate the effects and the underlying mechanisms of HDAC3 inhibition in cerebral I/R injury in diabetic state.

Cerebral I/R injury is a well-known clinical complication during the treatment of stroke, and there is no effective treatment in clinic [36]. Diabetes is one of the most important independent risk factors for the development of cerebrovascular disease. Studies have shown that diabetes can increase the risk of cerebrovascular disease by more than 4 times and increase the risk of stroke by 1.8 to 6 times [37]. Compared with nondiabetic patients, patients with diabetes are highly vulnerable to cerebral ischemia. In particular, after recovery of blood perfusion, diabetic patients with stroke have higher incidence of cerebral I/R injury, worse prognosis, and higher mortality compared with their nondiabetic counterparts [37, 38]. Evidences suggested that effective inhibition of HDAC3 may help to prevent or treat either ischemic stroke or diabetes, respectively [24, 39]. However, there are many questions remain to be further elucidated, especially regarding the role and the underlying mechanisms of HDAC3 in the pathophysiology of diabetes complicated with ischemic stroke.

The remarkable increase of reactive oxygen species (ROS) is one of the pathophysiological characteristics of diabetes [40]. ROS can not only directly cause oxidative damage of tissues and organs but also indirectly inhibit the activity of the endothelial nitric oxide synthase (eNOS) via the decoupling and activation of PKC-*β*, which induces the production of peroxynitrite anion (ONOO^−^) with stronger protein nitration ability and cytotoxicity, thereby aggravating the injury [41, 42]. It suggests that the enhancements of oxidative stress and the secondary damage caused by enhanced oxidative stress in diabetic state are probably the main reasons why the organs of diabetic patients are vulnerable to I/R injury. As expected, our findings demonstrated that excessive ROS production and redundant lipid peroxidation products (such as MDA), accompanied with insufficient antioxidants (such as SOD) in the brain tissue from the diabetic mice subjected to cerebral I/R, resulted in a severe brain injury.

Another important pathological mechanism of diabetes is autophagic dysfunction [43, 44]. Autophagy is a dynamic process that sequesters nonessential intracellular components into double-membraned autophagosomes for lysosomal degradation in response to various stress stimuli, including ROS aggregates, hyperglycemia, and accumulation of damaged molecules and organelles [29]. Both inadequate autophagy and excessive autophagy threaten cell metabolism and survival [45]. When the stress stimuli remain unresolved, continued autophagy activation progresses to autophagy defect which is associated with the development of metabolic syndrome, lipid abnormalities, and diabetes [40]. Evidence from our group [26, 45], as well as others [44, 46], have demonstrated that autophagy is inhibited in diabetic state. Autophagy also plays an important role in the pathogenesis of cerebral I/R injury [47]. Under nondiabetic states, I/R induces a burst production of ROS which can directly stimulate the formation of autophagosomes via oxidative modification of autophagy, thereby enhancing autophagy, effectively eliminating damaged organelles, and inhibiting endogenous apoptotic pathways [48]. However, our study found autophagy was just slightly, but not statistically, increased in the diabetic mice with I/R compared to the diabetic mice with the sham operation. The result was similar to the recent report in which autophagy exerts cardioprotective effects on myocardial I/R injury in normal mice, but not in diabetic mice [49]. Being chronically inhibited in diabetic state, autophagy cannot be effectively activated in diabetic stroke mice when compared to diabetic mice. This may explain why the autophagy indicators (LC3B, beclin-1, and P62) are not modified in DIR mice when compared to DS ones.

HDAC3 is a key regulator of energy metabolism and glucose metabolism [50, 51]. Suppression of HDAC3 enhances oxidative metabolism, regulates gluconeogenesis, reduces hyperglycaemia, and increases insulin secretion, which are conductive in improving diabetes [23]. The upregulation of HDAC3 in ischemic stroke mediates deleterious effects; specific inhibition of HDAC3 protects the brain against ischemic injury 24 hours after MCAO by initiating a series of gene expression programs associated with neuroprotection in animal models [20, 24]. In our study, we observed that in the diabetic stroke model, inhibition of HDAC3 by RGFP966 noticeably decreased the cerebral infarct volume, ameliorated pathological changes of brain tissues in vivo, and improved the viability and cytotoxicity in vitro. HDAC3 has been demonstrated to negatively regulate the transcription of the target gene Bmal1 which is implicated in the regulation of oxidative stress and autophagy [50–52]. Bmal1 can not only regulate tissue homeostasis via the modulation of reactive oxygen species (ROS) but also regulate the antioxidant defense via the control of major antioxidant enzymes [53, 54]. In our study, we used RGFP966 to inhibit the expression of HDAC3 in the diabetic mice subjected to cerebral I/R injury, which significantly increased the expression of Bmal1. Simultaneously, the level of ROS was dramatically decreased, and the activity of SOD was remarkably increased. Qiao et al. have proved that overexpression of Bmal1 alleviates the high glucose-induced suppression of autophagy, while this effect can be reversed by Bmal1 knockdown [46]. Similar to their study, our study found that HDAC3 was prominently increased in the diabetic mice subjected to cerebral I/R injury, which mediated a marked repression of Bmal1 in the brain tissues, accompanied by an inconspicuous change in autophagy. However, when the HDAC3 inhibitor was given, the expression of Bmal1 was significantly increased, coupled with the prominent upregulation of autophagy. This phenomenon was demonstrated by an increase in the autophagosomes' number, an upregulation of LC3B and beclin-1 expression, and a decrease in p62 expression in the diabetic mice subjected to cerebral I/R injury.

Obesity and diabetes are highly related to defects in oxidative metabolism. It has been reported that selective HDAC inhibition significantly enhances oxidative metabolism in skeletal muscle and adipose tissue and modulates energy expenditure in obese diabetic mice [55]. In addition, selective HDAC3 inhibition is demonstrated to prevent diabetes-induced liver damage and reduce diabetes-induced aorta pathologies by activating nuclear factor erythroid 2-related factor 2 [56]. The effects of HDAC3 inhibition on diabetic ischemic stroke have not been researched. Our study indicated that selective inhibition of HDAC3 exerts protective effects against cerebral I/R injury in diabetic state in vivo and in vitro via the modulation of oxidative stress, apoptosis, and autophagy, which might be mediated by the upregulation of Bmal1. It is prospective that HDAC3 inhibition may be a potential drug target for the treatment of diabetes complicated with ischemic stroke.

Recently, more and more attempts are made to treat diseases by using a plant-derived or indigenous compound. Traditional Chinese medicine has drawn great attention because of its relative safety and long history [57]. Tanshinone IIA (TIIA), derived from the rhizome of Salvia miltiorrhiza, is widely used as a quality control marker in Chinese pharmacopoeia. TIIA is found to decrease the expression levels and enzyme activity of HDAC1, HDAC3, HDAC4, and HDAC8 [58]. Apigenin largely exists in common vegetables and fruits. Apigenin is demonstrated to inhibit the expression of HDAC1 and HDAC3, as well as increasing the acetylation of histones H3 and H4 [59]. In addition, silymarin is a flavonoid extracted from milk thistle plant. Silymarin is showed to reduce the levels of HDAC1, HDAC2, HDAC3, and HDAC8 and increase the activity of HATs [60]. However, when compared with RGFP966, plant-derived compounds mentioned above lack specificity to one histone deacetylase. RGFP966, as a specific inhibitor for HDAC3, is more promising for the treatment of diabetes complicated with ischemic stroke.

Diabetes constitutes a major risk factor for ischemic stroke owing to its promotive effects on cerebrovascular vulnerability and susceptibility. Recent study identified that common genetic variants on chromosome 9p21 confer risk of ischemic stroke [61]. In addition, with the uncontrolled industrialization, people have been exposed to environmental pollutants unknowingly, continually, and chronically. Environmental pollutants, including PM2.5, microplastic pollutants, and bisphenol A, have been identified as new risk factors for diabetes [62, 63]. Our study demonstrated that HDAC3 inhibitor RGFP966 is a potential drug target for the treatment of diabetes complicated with ischemic stroke. However, it is well known that prevention comes first for all diseases. Environmental protection and lifestyle intervention should be taken into account for battling with diabetes and stroke.

There are several limitations in our study that need to be addressed. The HDAC3 inhibitor RGFP966 is not specific to the brain tissue, and the effects of RGFP966 in tissues other than brain tissues are not included in our study. In addition, the exact mechanisms in which autophagy is of no significant difference between DIR mice and DS mice have not been clarified. A further study is required.

## 5. Conclusions

In conclusion, our study suggested that the HDAC3 was implicated in the pathogenesis of the diabetic stroke. Suppression of HDAC3 exerted protective effects against cerebral I/R and H/R injury in diabetic state via the enhancement of oxidative stress, inhibition of apoptosis, and improvement of autophagy. The protective effects of HDAC3 inhibition might be mediated by the upregulation of Bmal1. It is believed that HDAC3 may become a potential drug target for the treatment of diabetes complicated with ischemic stroke.

## Data Availability

The data used to support the findings of this study are included within the article.

## References

[1] Liu L., Wang D., Wong K. S. L., Wang Y. (2011). Stroke and stroke care in China: huge burden, significant workload, and a national priority. *Stroke*.

[2] Schellinger P. D., Kohrmann M. (2014). 4.5-hour time window for intravenous thrombolysis with recombinant tissue-type plasminogen activator is established firmly. *Stroke*.

[3] Shou J., Zhou L., Zhu S., Zhang X. (2015). Diabetes is an independent risk factor for stroke recurrence in stroke patients: a meta-analysis. *Journal of Stroke & Cerebrovascular Diseases*.

[4] Osei E., den Hertog H. M., Berkhemer O. A. (2017). Admission glucose and effect of intra-arterial treatment in patients with acute ischemic stroke. *Stroke*.

[5] Pan Y., Jing J., Li H., Wang Y., Wang Y., He Y. (2016). Abnormal glucose regulation increases stroke risk in minor ischemic stroke or TIA. *Neurology*.

[6] Zimmet P., Alberti K. G., Magliano D. J., Bennett P. H. (2016). Diabetes mellitus statistics on prevalence and mortality: facts and fallacies. *Nature Reviews Endocrinology*.

[7] Yang L., Shao J., Bian Y. (2016). Prevalence of type 2 diabetes mellitus among inland residents in China (2000-2014): a meta-analysis. *Journal of Diabetes Investigation*.

[8] Park D. J., Koh P. O. (2018). Diabetes aggravates decreases in hippocalcin and parvalbumin expression in focal cerebral ischemia. *Neuroscience Letters*.

[9] Al-Rubeaan K., al-Hussain F., Youssef A. M., Subhani S. N., al-Sharqawi A. H., Ibrahim H. M. (2016). Ischemic stroke and its risk factors in a registry-based large cross-sectional diabetic cohort in a country facing a diabetes epidemic. *Journal of Diabetes Research*.

[10] Zhang X., Mu Y., Yan W., Ba J., Li H. (2014). Prevalence of stroke and metabolic disorders in the middle-aged and elderly Chinese with type 2 diabetes. *Chinese Medical Journal*.

[11] Chen J., Ye X., Yan T. (2011). Adverse effects of bone marrow stromal cell treatment of stroke in diabetic rats. *Stroke*.

[12] Venkat P., Chopp M., Chen J. (2018). Cell-based and exosome therapy in diabetic stroke. *Stem Cells Translational Medicine*.

[13] Basu Mallik S., Jayashree B. S., Shenoy R. R. (2018). Epigenetic modulation of macrophage polarization- perspectives in diabetic wounds. *Journal of Diabetes and its Complications*.

[14] Jhelum P., Karisetty B. C., Kumar A., Chakravarty S. (2017). Implications of epigenetic mechanisms and their targets in cerebral ischemia models. *Current Neuropharmacology*.

[15] Aune S. E., Herr D. J., Kutz C. J., Menick D. R. (2015). Histone deacetylases exert class-specific roles in conditioning the brain and heart against acute ischemic injury. *Frontiers in Neurology*.

[16] Park M. J., Sohrabji F. (2016). The histone deacetylase inhibitor, sodium butyrate, exhibits neuroprotective effects for ischemic stroke in middle-aged female rats. *Journal of Neuroinflammation*.

[17] Sun Z., Miller R. A., Patel R. T. (2012). Hepatic Hdac3 promotes gluconeogenesis by repressing lipid synthesis and sequestration. *Nature Medicine*.

[18] Sun X. Y., Qu Y., Ni A. R. (2017). Novel histone deacetylase inhibitor N25 exerts anti-tumor effects and induces autophagy in human glioma cells by inhibiting HDAC3. *Oncotarget*.

[19] Sathishkumar C., Prabu P., Balakumar M. (2016). Augmentation of histone deacetylase 3 (*HDAC3*) epigenetic signature at the interface of proinflammation and insulin resistance in patients with type 2 diabetes. *Clinical Epigenetics*.

[20] Chen Y. T., Zang X. F., Pan J. (2012). Expression patterns of histone deacetylases in experimental stroke and potential targets for neuroprotection. *Clinical and Experimental Pharmacology & Physiology*.

[21] Hong S., Zhou W., Fang B. (2017). Dissociation of muscle insulin sensitivity from exercise endurance in mice by HDAC3 depletion. *Nature Medicine*.

[22] Zampetaki A., Zeng L., Margariti A. (2010). Histone deacetylase 3 is critical in endothelial survival and atherosclerosis development in response to disturbed flow. *Circulation*.

[23] Meier B. C., Wagner B. K. (2014). Inhibition of HDAC3 as a strategy for developing novel diabetes therapeutics. *Epigenomics*.

[24] Yang X., Wu Q., Zhang L., Feng L. (2016). Inhibition of histone deacetylase 3 (HDAC3) mediates ischemic preconditioning and protects cortical neurons against ischemia in rats. *Frontiers in Molecular Neuroscience*.

[25] Zhang Y., Su W., Zhang Q. (2018). Glycine protects H9C2 cardiomyocytes from high glucose- and hypoxia/reoxygenation-induced injury via inhibiting PKC*β*2 activation and improving mitochondrial quality. *Journal of Diabetes Research*.

[26] Zhan L., Zhang Y., Su W. (2018). The roles of autophagy in acute lung injury induced by myocardial ischemia reperfusion in diabetic rats. *Journal of Diabetes Research*.

[27] Li P. C., Liu L. F., Jou M. J., Wang H. K. (2016). The GLP-1 receptor agonists exendin-4 and liraglutide alleviate oxidative stress and cognitive and micturition deficits induced by middle cerebral artery occlusion in diabetic mice. *BMC Neuroscience*.

[28] Rana A. K., Singh D. (2018). Targeting glycogen synthase kinase-3 for oxidative stress and neuroinflammation: opportunities, challenges and future directions for cerebral stroke management. *Neuropharmacology*.

[29] Sun Y., Zhang T., Zhang Y. (2018). Ischemic postconditioning alleviates cerebral ischemia–reperfusion injury through activating autophagy during early reperfusion in rats. *Neurochemical Research*.

[30] Malvaez M., McQuown S. C., Rogge G. A. (2013). HDAC3-selective inhibitor enhances extinction of cocaine-seeking behavior in a persistent manner. *Proceedings of the National Academy of Sciences of the United States of America*.

[31] Zhao B., Liu L., Leng Y. (2017). The role of histone deacetylase inhibitors in regulation of Akt/GSK-3*β* signaling pathway in mice following transient focal cerebral ischemia. *Acta Cirúrgica Brasileira*.

[32] Wu X., Liu X., Huang H. (2018). Effects of major ozonated autoheamotherapy on functional recovery, ischemic brain tissue apoptosis and oxygen free radical damage in the rat model of cerebral ischemia. *Journal of Cellular Biochemistry*.

[33] Sun L. N., Shen J., Su F. (2009). Bicyclol attenuates oxidative stress and neuronal damage following transient forebrain ischemia in mouse cortex and hippocampus. *Neuroscience Letters*.

[34] Lan R., Wu J. T., Wu T. (2018). Mitophagy is activated in brain damage induced by cerebral ischemia and reperfusion via the PINK1/Parkin/p62 signalling pathway. *Brain Research Bulletin*.

[35] Kobayashi S., Xu X., Chen K., Liang Q. (2012). Suppression of autophagy is protective in high glucose-induced cardiomyocyte injury. *Autophagy*.

[36] Lu C., Ha T., Wang X. (2014). The TLR9 ligand, CpG-ODN, induces protection against cerebral ischemia/reperfusion injury via activation of PI3K/Akt signaling. *Journal of the American Heart Association*.

[37] Kalani A., Kamat P. K., Tyagi N. (2015). Diabetic stroke severity: epigenetic remodeling and neuronal, glial, and vascular dysfunction. *Diabetes*.

[38] Yan T., Venkat P., Chopp M. (2015). Neurorestorative therapy of stroke in type 2 diabetes mellitus rats treated with human umbilical cord blood cells. *Stroke*.

[39] Lundh M., Galbo T., Poulsen S. S., Mandrup-Poulsen T. (2015). Histone deacetylase 3 inhibition improves glycaemia and insulin secretion in obese diabetic rats. *Diabetes, Obesity and Metabolism*.

[40] Muriach M., Flores-Bellver M., Romero F. J., Barcia J. M. (2014). Diabetes and the brain: oxidative stress, inflammation, and autophagy. *Oxidative Medicine and Cellular Longevity*.

[41] Ramdial K., Franco M. C., Estevez A. G. (2017). Cellular mechanisms of peroxynitrite-induced neuronal death. *Brain Research Bulletin*.

[42] Wilhelm J., Vytasek R., Uhlik J., Vajner L. (2016). Oxidative stress in the developing rat brain due to production of reactive oxygen and nitrogen species. *Oxidative Medicine and Cellular Longevity*.

[43] Fetterman J. L., Holbrook M., Flint N. (2016). Restoration of autophagy in endothelial cells from patients with diabetes mellitus improves nitric oxide signaling. *Atherosclerosis*.

[44] Feidantsis K., Mellidis K., Galatou E., Sinakos Z., Lazou A. (2018). Treatment with crocin improves cardiac dysfunction by normalizing autophagy and inhibiting apoptosis in STZ-induced diabetic cardiomyopathy. *Nutrition, Metabolism & Cardiovascular Diseases*.

[45] Zhou B., Lei S., Xue R., Leng Y., Xia Z., Xia Z. Y. (2017). DJ-1 overexpression restores ischaemic post-conditioning-mediated cardioprotection in diabetic rats: role of autophagy. *Clinical Science*.

[46] Qiao L., Guo B., Zhang H. (2017). The clock gene, brain and muscle Arnt-like 1, regulates autophagy in high glucose-induced cardiomyocyte injury. *Oncotarget*.

[47] Deng Y. H., He H. Y., Yang L. Q., Zhang P. Y. (2016). Dynamic changes in neuronal autophagy and apoptosis in the ischemic penumbra following permanent ischemic stroke. *Neural Regeneration Research*.

[48] Fang C., Gu L., Smerin D., Mao S., Xiong X. (2017). The interrelation between reactive oxygen species and autophagy in neurological disorders. *Oxidative Medicine and Cellular Longevity*.

[49] Han Z., Cao J., Song D. (2014). Autophagy is involved in the cardioprotection effect of remote limb ischemic postconditioning on myocardial ischemia/reperfusion injury in normal mice, but not diabetic mice. *PLoS One*.

[50] Emmett M. J., Lim H. W., Jager J. (2017). Histone deacetylase 3 prepares brown adipose tissue for acute thermogenic challenge. *Nature*.

[51] Shi G., Xie P., Qu Z. (2016). Distinct roles of HDAC3 in the core circadian negative feedback loop are critical for clock function. *Cell Reports*.

[52] Dong C., Gongora R., Sosulski M. L., Luo F., Sanchez C. G. (2016). Regulation of transforming growth factor-beta1 (TGF-*β*1)-induced pro-fibrotic activities by circadian clock gene BMAL1. *Respiratory Research*.

[53] Khapre R. V., Kondratova A. A., Susova O., Kondratov R. V. (2011). Circadian clock protein BMAL1 regulates cellular senescence in vivo. *Cell Cycle*.

[54] Kondratov R. V., Vykhovanets O., Kondratova A. A., Antoch M. P. (2009). Antioxidant N-acetyl-L-cysteine ameliorates symptoms of premature aging associated with the deficiency of the circadian protein BMAL1. *Aging*.

[55] Galmozzi A., Mitro N., Ferrari A. (2013). Inhibition of class I histone deacetylases unveils a mitochondrial signature and enhances oxidative metabolism in skeletal muscle and adipose tissue. *Diabetes*.

[56] Zhang J., Xu Z., Gu J. (2018). HDAC3 inhibition in diabetic mice may activate Nrf2 preventing diabetes-induced liver damage and FGF21 synthesis and secretion leading to aortic protection. *American Journal of Physiology-Endocrinology and Metabolism*.

[57] Xu Q., Bauer R., Hendry B. M. (2013). The quest for modernisation of traditional Chinese medicine. *BMC Complementary and Alternative Medicine*.

[58] Wang L., Zhang C., Guo Y. (2014). Blocking of JB6 cell transformation by tanshinone IIA: epigenetic reactivation of Nrf2 antioxidative stress pathway. *The AAPS Journal*.

[59] Pandey M., Kaur P., Shukla S., Abbas A., Fu P., Gupta S. (2012). Plant flavone apigenin inhibits HDAC and remodels chromatin to induce growth arrest and apoptosis in human prostate cancer cells: in vitro and in vivo study. *Molecular Carcinogenesis*.

[60] Singh T., Prasad R., Katiyar S. K. (2016). Therapeutic intervention of silymarin on the migration of non-small cell lung cancer cells is associated with the axis of multiple molecular targets including class 1 HDACs, ZEB1 expression, and restoration of miR-203 and E-cadherin expression. *American Journal of Cancer Research*.

[61] Smith J. G., Melander O., Lövkvist H. (2009). Common genetic variants on chromosome 9p21 confers risk of ischemic stroke: a large-scale genetic association study. *Circulation: Cardiovascular Genetics*.

[62] Bowe B., Xie Y., Li T., Yan Y., Xian H., al-Aly Z. (2018). The 2016 global and national burden of diabetes mellitus attributable to PM_2·5_ air pollution. *The Lancet Planetary Health*.

[63] Jeon J. Y., Ha K. H., Kim D. J. (2015). New risk factors for obesity and diabetes: environmental chemicals. *Journal of Diabetes Investigation*.

